# Watching the Operations of Nature

**Published:** 1884-07

**Authors:** 


### HALL'S JOURNAL OF HEALTH

—AND—

**MEDICINE.**

**MONTHLY.**

E. H. GIBBS, A. M., M. D., EDITOR.

FOUNDED IN 1854.

### WATCHING THE OPERATIONS OF NATURE.

ONLY physicians of large experience have an adequate conception of the mischief to health and happiness that results through watching the complicated machinery of one's own system, in its mysterious operations. Health is the natural condition of all living creatures; and Nature's remedies are the best for restoring to their normal condition such functions as may become deranged through sudden changes of temperature, or other ordinary causes. The old saying, that "a little knowledge is a dangerous thing," is especially applicable to that large class of individuals who think themselves afflicted, more or less, with some malady that they believe very common and very fatal; their ideas having been almost unconsciously formed from glancing day after day at some flaming advertisements headed, CONSUMPTION, KIDNEY DISEASE, LIVER COMPLAINT, HEART DISEASE, &c., &c. These advestisements show great ingenuity, and are well calculated to excite the fears of nervous persons. There are times when a man in perfect health will present symptoms peculiar to all of these diseases. Few individuals have lived to middle age without having experienced some of the symptoms of consumption: as cough with suspicious expectoration sometimes streaked with blood, pain in the chest and even "night sweats" and apparently "hectic fever." These often result from a cold, and pass off in a few days, leaving not a vestige of any disease. Symptoms of liver complaint and heart disease almost invariably attend all grades of dyspepsia. Over fatigue or exhaustion from any cause, but more particularly from nervous depression caused by grief and anxiety, is almost invariably attended by palpitation of the heart. This symptom quickly subsides as the strength returns, unless the imagination magnifies it into actual disease, by secretly brooding over it. But of diseases that haunt nervous people at the present day, those that relate to affections of the kidneys are the most common in imagination, and perhaps the most seldom met with in actual practice. Every one knows that kidney disease is in some way connected with an unnatural condition of the secretions; and it seems wonderfully easy to detect disease by watching these secretions. At times the flow is abundant and clear, and then scanty and highly-colored, leaving a sediment at the bottom of the vessel when standing a few hours. The nervous man sees cause for alarm. He purchases a generous supply of some famous "kidney cure," reads the circulars, which confirm him in the belief that his is a desperate case; takes the remedy and in due time finds himself restored to health—"a brand plucked from the burning,"—and he gladly gives his certificate to the supposed facts; the actual facts being that he would have recovered just as soon by a proper regulation of his diet, and that if kidney disease had really existed, the "kidney remedy" would have had no more effect upon it than moon-beams on an iceberg. There is no disease so surely destructive as doubt and fear; and no remedy so potent as that which works upon the imagination. It is usually best to know the worst, but in more than half the cases of doubt an examination by a competent physician would prove either that no actual disease existed, or that it would yield to timely remedies if it did exist.

Not only are the secretions affected by the character and quantity of the various foods and drinks taken into the stomach, but by the various conditions of the atmosphere, as to heat and cold, dryness and dampness. But the most powerful agent in changing the secretions is nervous influence. There is usually a scanty flow, with high color, when, grief, anxiety, fear or sorrow exist; but there is seldom any actual disease, certainly not from any of these causes, unless of long continuance. Finally, it may be said that many of the affections that people think they are afflicted with are simply temporary disturbances that disappear by attention to the diet and a return of mental tranquility.

